# Proteolytic Degradation of reduced Human Beta Defensin 1 generates a Novel Antibiotic Octapeptide

**DOI:** 10.1038/s41598-019-40216-2

**Published:** 2019-03-06

**Authors:** Judith Wendler, Bjoern O. Schroeder, Dirk Ehmann, Louis Koeninger, Daniela Mailänder-Sánchez, Christina Lemberg, Stephanie Wanner, Martin Schaller, Eduard F. Stange, Nisar P. Malek, Christopher Weidenmaier, Salomé LeibundGut-Landmann, Jan Wehkamp

**Affiliations:** 1 Department of Internal Medicine 1, University Hospital Tuebingen, Tuebingen, Germany; 2 Dr. Margarete Fischer-Bosch-Institute of Clinical Pharmacology, Stuttgart and University of Tuebingen, Tuebingen, Germany; 3 Institute of Immunology, Vetsuisse Faculty, University of Zürich, Zurich, Switzerland; 4 Institute of Dermatology, University Hospital Tuebingen, Tuebingen, Germany; 5 Institute of Medical Microbiology and Hygiene, University Hospital Tuebingen, Tuebingen, Germany; 6 Present Address: Wallenberg Laboratory, University of Gothenburg, Gothenburg, Sweden; 7 Present Address: Institute of Immunology, Vetsuisse Faculty, University of Zürich, Zurich, Switzerland

## Abstract

Microbial resistance against clinical used antibiotics is on the rise. Accordingly, there is a high demand for new innovative antimicrobial strategies. The host-defense peptide human beta-defensin 1 (hBD-1) is produced continuously by epithelial cells and exhibits compelling antimicrobial activity after reduction of its disulphide bridges. Here we report that proteolysis of reduced hBD-1 by gastrointestinal proteases as well as human duodenal secretions produces an eight-amino acid carboxy-terminal fragment. The generated octapeptide retains antibiotic activity, yet with distinct characteristics differing from the full-length peptide. We modified the octapeptide by stabilizing its termini and by using non-natural D-amino acids. The native and modified peptide variants showed antibiotic activity against pathogenic as well as antibiotic-resistant microorganisms, including *E*. *coli*, *P*. *aeruginosa* and *C*. *albicans*. Moreover, in an *in vitro C*. *albicans* infection model the tested peptides demonstrated effective amelioration of *C*. *albicans* infection without showing cytotoxity on human cells. In summary, protease degradation of hBD-1 provides a yet unknown mechanism to broaden antimicrobial host defense, which could be used to develop defensin-derived therapeutic applications.

## Introduction

Antimicrobial peptides (AMPs) are evolutionary ancient peptide antibiotics produced by all multicellular organisms. They are part of the primary defense against microbial infections and exhibit antimicrobial activity against bacteria, fungi and some enveloped viruses^1,2^. Humans produce different classes of AMPs, one of them are the defensins. These secreted peptides are characterized by their small size (3 to 5 kDa), cationic net charge and six conserved cysteine residues, which are connected via three disulphide bridges^1,3,4^. Human beta-defensin 1 (hBD-1) was the first beta-defensin identified in humans and is produced by epithelia, monocytes, plasmacyoid dendritic cells and platelets^5–8^. In contrast to inducible beta-defensins 2 and 3, hBD-1 is produced constitutively and its expression can be regulated by peroxisome proliferator-activated receptor gamma (PPARγ) and hypoxia-inducible factor alpha (HIF1-α)^6,9,10^. We could recently show that antimicrobial activity of hBD-1 is strongly increased after reduction of its three disulphide bridges independent of bacterial Gram-status^11,12^. Activation of the peptide could be executed by a reducing environment or enzymatically by the oxido-reductase thioredoxin^11,13^.

Due to their ancient evolutionary origin and the strong demand for novel antimicrobial strategies, AMPs have been considered as potential antibiotic drug candidates. Mainly because they target “Achilles heels” of microorganisms, only few resistance mechanisms have been evolved over long time^14,15^. Still, large-scale chemical synthesis of defensins containing three native disulphide-bridges has been a challenge and rendered the production expensive. Accordingly, the production of smaller, but yet antibiotic, fragments of defensins without disulphide bridges is a promising option. We have shown previously that reduced hBD-1 can be degraded by the intestinal protease trypsin^16^. Here, we evaluate a carboxy-terminal fragment of hBD-1 that is generated after proteolytic digestion by intestinal proteases. We investigate its antimicrobial activity and its potential to be exploited as a possible candidate for future antibiotic drug development.

## Results

### Degradation of reduced hBD-1 generates an antimicrobial octapeptide

**Figure 1:**
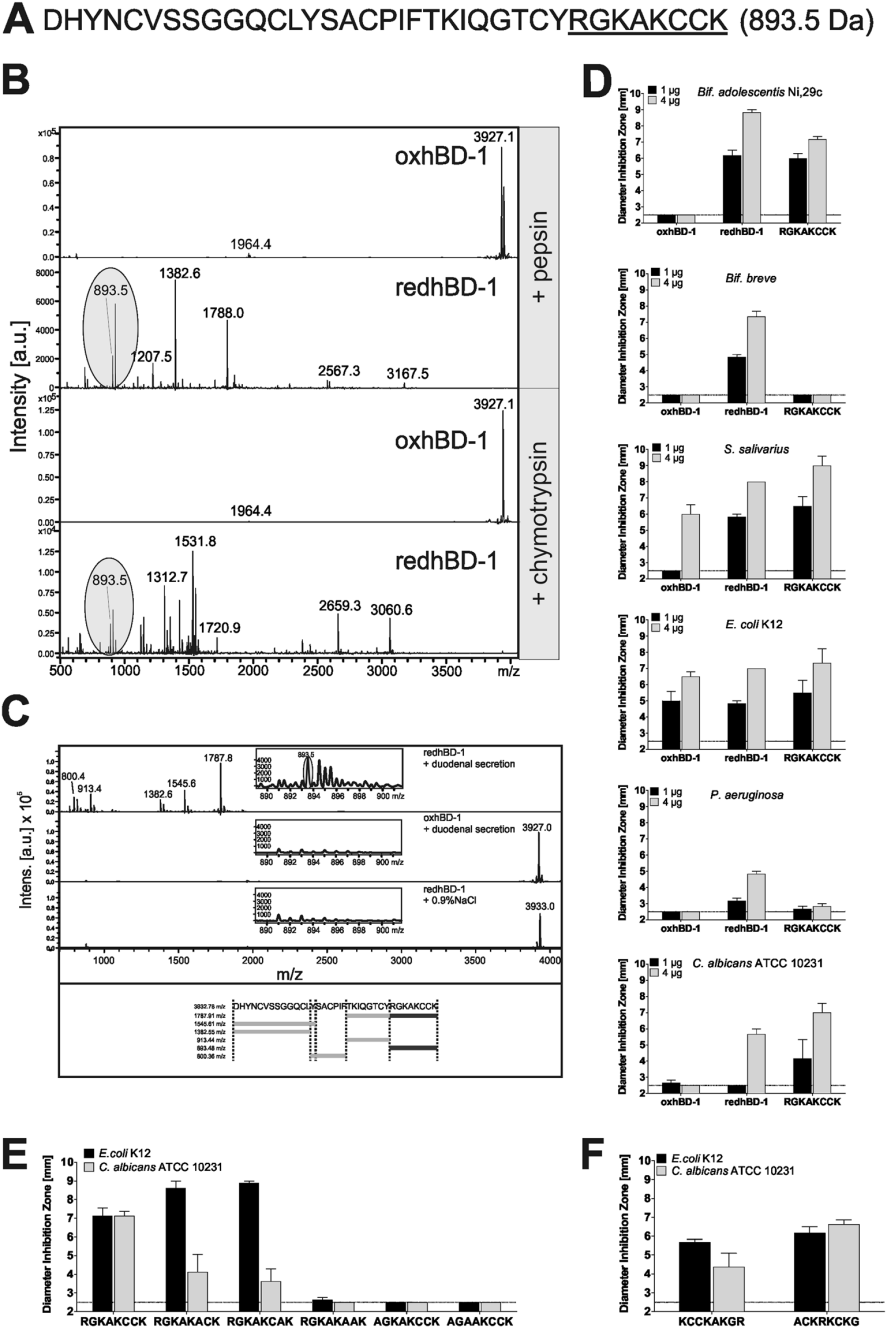
Proteolytic digestion of reduced hBD-1 generates an antimicrobial octapeptide. (**A**) Aminoacid sequence of hBD-1, depicted as one-letter code. (**B**) The oxidized and the reduced form of hBD-1 were digested with pepsin or chemotrypsin and fragments were analyzed by MALDI-MS. The carboxy-terminal fragment RGKAKCCK (893.5 Da, highlighted in (**A**) was further investigated. (**C**) The oxidized and the reduced hBD-1 were digested with human duodenal secretion and fragments were analyzed by MALDI-MS. Fragments were identified by comparison with an *in silico* digest using ExPASy software. (**D**) Different peptide concentrations of oxidized (ox) and reduced (red) as well as the carboxy-terminal octapeptide RGKAKCCK were tested in an antimicrobial diffusion assay against several microbial strains. Diameter of inhibition zones indicates antimicrobial activity; a diameter of 2.5 mm (dotted line) is the diameter of an empty well. (**E**,**F**) Modifications of RGKAKCCK (4 µg) were tested in an antimicrobial diffusion assay against *E*. *coli* and a fungal strain *C*. *albicans*. Letters indicate amino acid one-letter code. All diffusion assays were carried out at least three times, mean +/− SEM is shown.

Reduction of the three disulphide bridges of hBD-1 yields a linear peptide which not only differs structurally from the oxidized form^11^, but is also more prone to proteolytic degradation by the protease trypsin^16^. To further analyze proteolytic susceptibility of hBD-1 (Fig. 1A) towards physiological gastro-intestinal proteases, we treated oxidized and reduced hBD-1 with pepsin and chymotrypsin (Fig. 1B). Similar to trypsin digestion, oxidized hBD-1 was protease resistant while the reduced form was readily digested. Focusing on the degradation products we detected a fragment having an m/z of 893.5, corresponding to the eight carboxy-terminal amino acids of hBD-1, NH_2_-RGKAKCCK-COOH (RGKAKCCK). To assess the *in vivo* relevance of our findings in more detail, we incubated oxidized and reduced hBD-1 with human duodenal secretion, which is rich in proteolytic enzymes. In agreement with our *in vitro* data, *ex vivo* digestion generated a fragment having an m/z of 893.5 (Fig. 1C) for reduced hBD-1 but not for oxidized hBD-1. While the *in vivo* presence of the octapeptide in the human gut remains to be proven, this finding supports the hypothesis that proteolytic cleavage of reduced hBD-1 could generate a novel antimicrobial peptide in the human intestine. To test whether antibiotic activity is retained in this degradation product we next analyzed antimicrobial activity of the terminal octapeptide. In a radial diffusion assay (RDA)^17^ we thus compared activity of oxidized and reduced hBD-1 with the octapeptide RGKAKCCK against selected commensal and pathogenic microorganisms (Fig. 1D). The octapeptide exhibited convincing activity against *Bifidobacterium adolescentis*, *Streptococcus salivarius ssp*. *thermophilus*, *Escherichia coli*, *Candida albicans* but neglectable antibiotic activity against *Pseudomonas aeruginosa*. While RGKAKCCK and oxidized hBD-1 generated no inhibition zones against *Bifidobacterium breve*, only reduced hBD-1 inhibited its growth. As expected, however, semi-quantitative evaluation on a molar base (4 µg reduced hBD-1 is equivalent to 254 µM in the RDA while 1 µg of octapeptide is equivalent to 280 µM) revealed that activity of the isolated terminus is less potent compared with the full length-peptide (Fig. 1D).

We found previously that cysteine residues are crucial for antimicrobial activity of hBD-1 against *E*. *coli* and *Bif*. *adolescentis*^11,12^. We confirmed these results for the terminal octapeptide, as replacing both cysteines (RGKAKAAK) completely abolished activity against *E*. *coli and C*. *albicans* (Fig. 1E, Supplementary Fig. 1). However, replacing either Cys_6_ (RGKAKACK) or Cys_7_ (RGKAKCAK) by alanine resulted in strongly decreased activity against *C*. *albicans*, while antibiotic activity of those peptides increased against *E*. *coli*. Consequently, cysteine residues seem to have an important, yet different, role for the antibiotic mechanism against the tested fungi and bacteria.

As antimicrobial peptide activity also relies on a positive net charge^18,19^ we further investigated the role of positively charged amino acids (Fig. 1E). Despite having two cysteine residues, the variants lacking Arg_1_ (AGKAKCCK, net charge +3) or Arg_1_ and Lys_3_ (AGAAKCCK, net charge +2) were completely inactive against both tested microorganism. Thus, antimicrobial activity of the octapeptide RGKAKCCK (net charge +4) depends on cysteine residues as well as a particular positive charge. Yet, as a reversed-order peptide had lower activity than the wild-type peptide or a scrambled version, especially against *C*. *albicans* (Fig. 1F), not only the amino acid composition but also its sequential order or its position seem to be involved in the peptides’ activity.

### Characterization of RGKAKCCK and its modified variants

Since the discovery of antimicrobial peptides there is anticipation to exploit them as antibiotic drugs^20^. To test the potential of our octapeptide to be used as a therapeutic agent, we first generated peptide variants to improve its stability. To prevent non-specific cleavage by amino-carboxypeptidases, we chemically stabilized its termini by amino-terminal acetylation and carboxy-terminal amidation (Ac-RGKAKCCK-NH_2_) and generated both peptides also in D-amino acid configuration (rGkakcck and Ac-rGkakcck-NH_2_, respectively).

**Figure 2:**
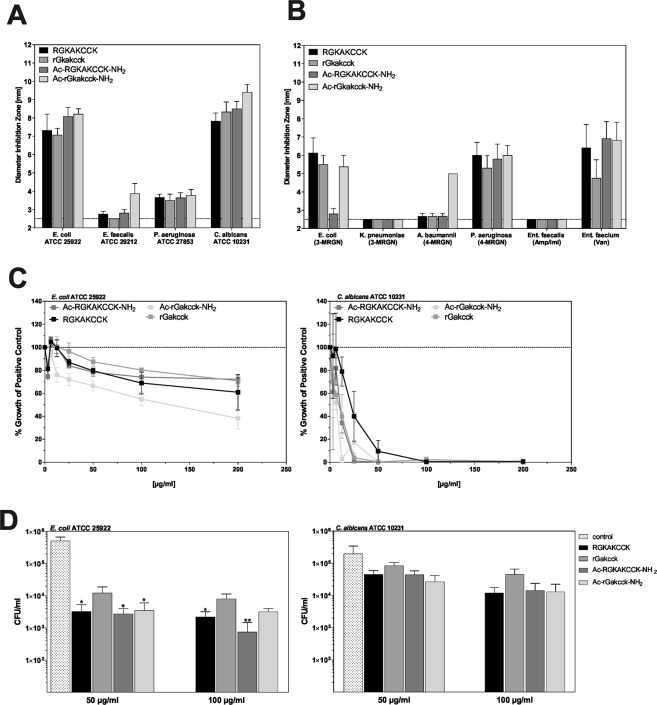
Antimicrobial activity of RGKAKCCK and its modified variants. The carboxyterminal octapeptide RGKAKCCK was stabilized at its termini by acetylation of the amino-terminus and amidation of the carboxy-terminus (Ac-RGKAKCCK-NH_2_). Both variants were also synthesized by using D-stereoisomeric amino acids (indicated by small letters). Antimicrobial activity of octapeptides (4 µg) was tested against pathogenic microorganisms of clinical relevance (**A**) and with antibiotic resistance (**B**) in radial diffusion assay. 3-MRGN: multi-resistant Gram negative pathogen (3 out of 4 antibiotic classes), 4-MRGN: multi-resistant Gram negative pathogen (4 out of 4 antibiotic classes) MRSA: Methicillin-resistant Staphylococcus aureus, Amp/Imi: Ampicillin/Imipinem, Van: Vancomycin. (**C**) Different concentrations of octapeptides were tested in a turbidity liquid assay against *E*. *coli* ATCC 25922 and *C*. *albicans* ATCC 10231. Peptides were incubated with tested microorganisms and change in optical density (OD_600nm_) was measured and % growth of untreated control was plotted after 12 hours. (**D**) Aliquots were plated on agar plates and colony forming units (CFUs) were calculated the next day. Data are presented as mean +/− SEM of at least three independent experiments. The statistical significance was evaluated by using Kruskal-Wallis test compared to control and marked with *p < 0.05 and **p < 0.01.

Next, to evaluate the antibiotic activity, we tested wild-type and modified peptide variants in their ability to inhibit growth of (opportunistic) pathogenic microorganisms (Fig. 2A). Direct comparison revealed that those variants with stabilized termini had promising activity against *E*. *coli* and *C*. *albicans*. Moreover, while all peptides displayed antimicrobial activity at pH 7.4, a reducing environment (DTT), acidification (pH 5.7), or a combination of acidification and reducing conditions (pH 5.7 + DTT) strongly decreased antimicrobial activity against *E*. *coli* (Supplementrary Fig. 2a) and *C*. *albicans* (Supplementrary Fig. 2b). Thus, antimicrobial acitivity of the generated octapeptides can be influenced by environmental conditions, in particular by reducing conditions with an acidic pH.

The widespread use of antibiotics in agriculture and to treat bacterial infections has led to a rapid emergence of microbial resistance^21,22^. As a consequence, in hospitals several multi-drug resistant strains exist that threaten effective therapy of microbial infections^23^. We therefore tested if the hBD1-derived peptide and its modified forms are also active against drug-resistant clinical isolates. As shown in Fig. 2B, we identified antimicrobial activity against clinical isolates of antibiotic-resistant *E*. *coli*, *Pseudomonas aeruginosa* and *Enterococcus faecium*. In contrast, *Acinetobacter baumanii* was only susceptible towards Ac-rGkakcck-NH_2_ whereas *Enterococcus faecalis* and *K*. *pneumoniae* were not sensitive. Direct comparison of the tested peptide revealed that the peptides RGKAKCCK and Ac-rGkakcck-NH_2_ inhibited growth of the most tested antibiotic-resistant bacteria, making them the most promising candidates for further drug development among the four tested peptides.

As the radial diffusion assay does not differentiate between microbistatic and microbicidal activity and contains immobilized bacteria or fungal cells, we complemented our antimicrobial tests with a broth microdilution assay to investigate susceptibility of *E*. *coli* and *C*. *albicans*. The tested octapeptides completely inhibited *C*. *albicans* growth at concentrations of 100 µg/ml, while the growth of *E*. *coli* was only inhibited to 40% of the untreated control by Ac-rGkakcck-NH_2_ (Fig. 2C). In addition, microbial cultures were incubated with the different peptides and colony forming units (CFU) were determined. For *E*. *coli* incubated with 100 µg/ml of RGKAKCCK or Ac-RGKAKCCK-NH_2,_ a more than 100-fold decrease in CFU was observed when compared to untreated controls (Fig. 2D). In contrast, CFU reduction of *C*. *albicans* was less pronounced, thus, indicating bactericidal activity against *E*. *coli* and a combination of fungicidal and fungistatic activity against *C*. *albicans* of the tested octapeptides.

**Figure 3:**
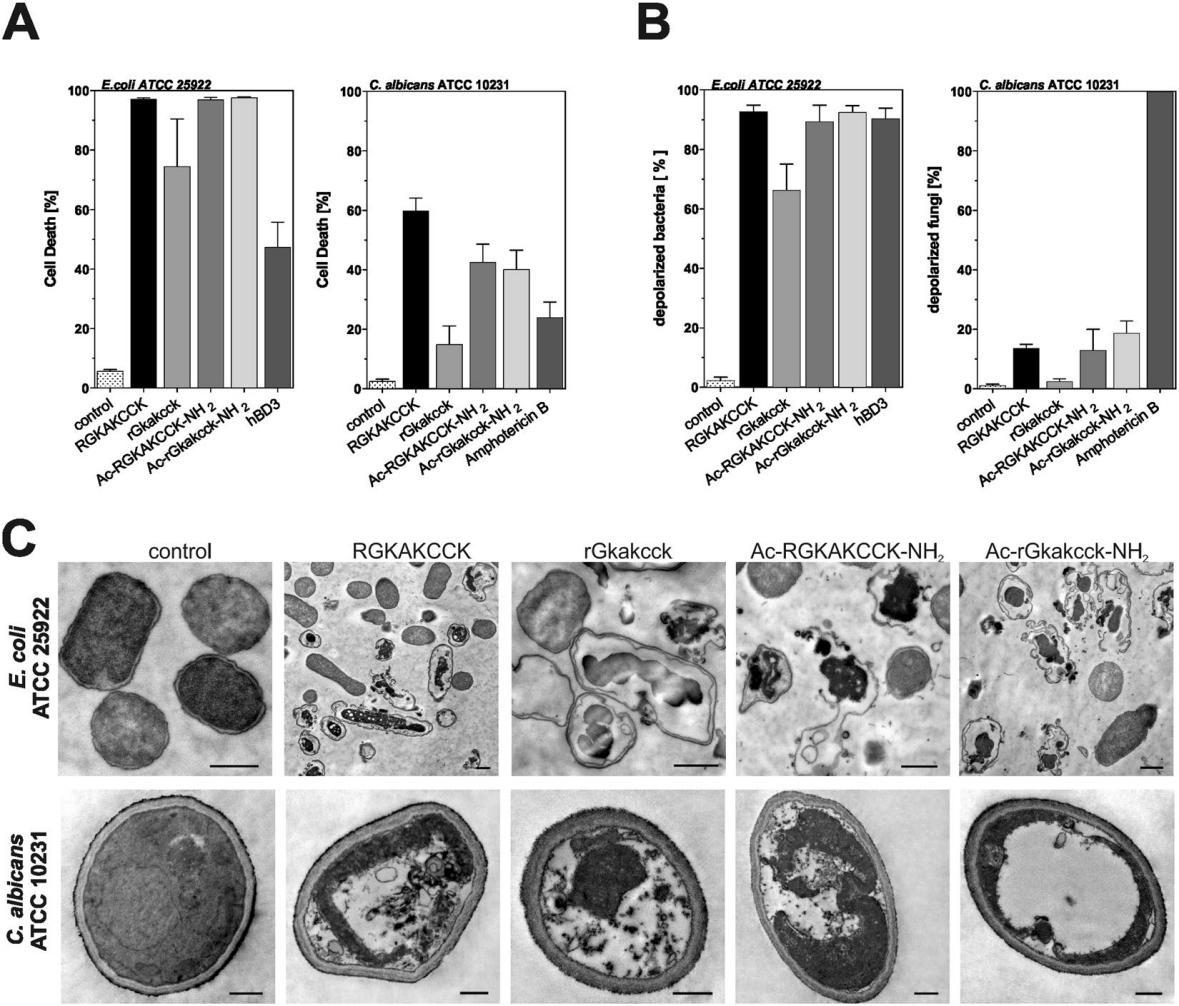
Characterization of mode of action in *E*. *coli* and *C*. *albicans*. (**A**) Membrane pores or (**B**) Membrane depolarization of 1 × 10^6^ CFU *E*. *coli* ATCC 25922 or *C*. *albicans* ATCC 10231 in response to 100 µg/ml octapeptides were tested. Microorganisms were treated 1 h with 100 µg/ml peptide and living organisms were analyzed by flow cytometry. As control we used hBD3 (50 µg/ml) and Amphotericin B (20 µg/ml) and untreated strains. Data are presented as mean +/− SEM of at least three independent experiments. (**C**) Transmission electron microcopy of *E*. *coli* (upper panel) and *C*. *albicans* (lower panel) treated with 400 µg/ml peptide. Magnification bar: 0.5 µm.

Many antimicrobial peptides target the microbial membrane^24,25^. To test whether this is also true for the octapeptides, we used a flow cytometric assay measuring membrane permeability by the dye propidium iodide (PI), which cannot permeate intact membranes. *E*. *coli* and *C*. *albicans* were incubated with the peptides and PI uptake, which indicated cell death, was analysed. For *E*. *coli*, all tested peptides exhibited at least 70% bacterial killing, while treatment with RGKAKCCK, Ac-RGKAKCCK-NH_2_ or Ac-rGkakcck-NH_2_ led to almost 100% cell death (Fig. 3A). In contrast, only the wild-type peptide RGKAKCCK induced up to 60% PI uptake when incubated with *C*. *albicans*.

In addition to PI uptake, we analyzed cellular membrane potential by using the membrane potential sensitive dye DiBAC_4_(3) (Fig. 3B). When incubating the peptides with *E*. *coli*, we observed strong membrane depolarization for the same peptides that caused PI uptake. In contrast, *C*. *albicans* displayed less than 20% of membrane depolarization. Thus, our results support a bactericidal effect of RGKAKCCK and Ac-rGkakcck-NH_2_ against *E*. *coli* by targeting the bacterial membrane, while the antibiotic effect against *C*. *albicans* seems rather membrane-independent.

To further investigate whether octapeptide treatment leads to structural damage of the microorganisms we used transmission electron microscopy (TEM) to visualize peptide-treated bacteria and fungi. Incubation of *E*. *coli* and *C*. *albicans* with all tested variants of the octapeptide led to different degrees of structural disintegration (Fig. 3C). This was especially pronounced in *E*. *coli*, where all peptides caused detachment of the cell membrane from the cytosol, cell wall and membrane disruption as well as disintegration of cytosolic structures. In *C*. *albicans*, however, no destruction of the fungal cell wall could be observed, confirming results from the flow cytometric expriments (Fig. 3A,B). Still, disintegration of cytosolic structures could also be observed in fungal cells, similar to the damage observed in *E*. *coli*. Taken together, our results demonstrate a bactericidal and fungicidal/fungistatic effect of the tested octapeptides.

### Potential of the octapeptides for therapeutic drug development

**Figure 4:**
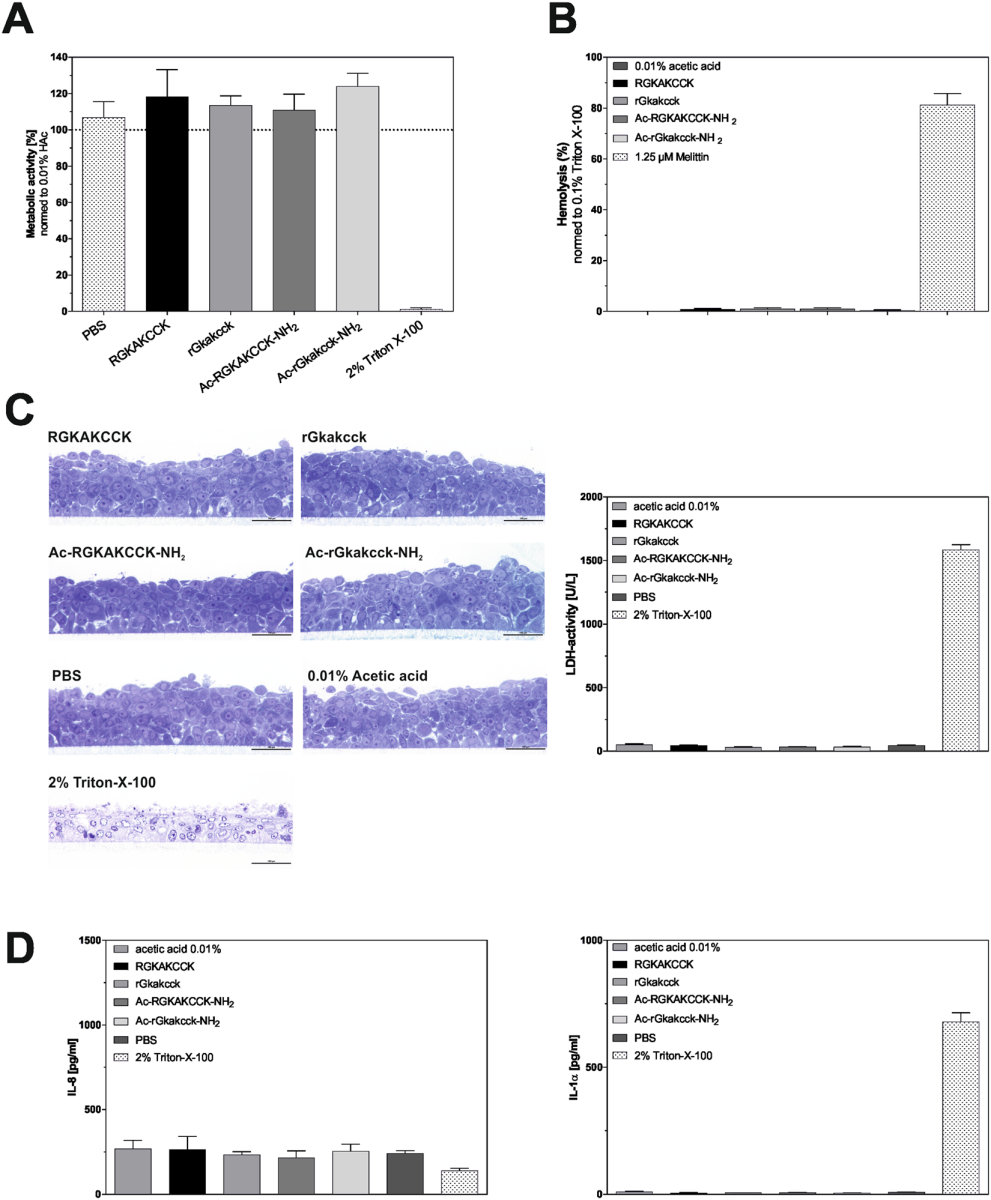
Modified octapeptides are not cytotoxic. Cytotoxicity of octapeptides (100 µg/ml) was investigated by (**A**) WST-1 based test against the human intestinal epithelial cell lines CaCo-2 and (**B**) Hemolytic Activity assay against erythrocytes, using 2% Trition-X-100 and 1.25 µM Melittin as positive control. (**C**) Histological analysis of model oral epithelia treated with 100 µg/ml peptide and cytotoxicity was additionally tested by lactatdehydrogenase release against this model human oral epithelium. (**D**) Cytokine release of model oral epithelia was analyzed by ELISA. Mean +/− SEM of three independent experiments is shown.

For potential therapeutic application, toxicity of the peptides needs to be excluded. We therefore analyzed cell-toxicity of all peptide variants against the intestinal epithelial cell line CaCo-2 and against erythrocytes. By utilization of a WST viability test we could not observe any cell toxicity in the relevant antimicrobial concentration ranges of 100 µg/ml (Fig. 4A) and 200 µg/ml (Supplementrary Fig. 4a) or any hemolytic effect against erythrocytes (Fig. 4B and Supplementrary Fig. 4b).

We furthermore tested the toxicity of the octapeptides by using *in vitro* reconstituted human oral epithelium (RHOE), which was analyzed by histology and lactatdehydrogenase (LDH) activity (Fig. 4C). No LDH release was detected with peptide variants, whereas the cytotoxic compound Triton-X-100, used as a positive control, induced a strong release of LDH. Similarly, when quantifying the release of the pro-inflammatory cytokines Interleukin-8 (IL-8) and IL-1α after incubation of the RHOE with the octapeptides we did not detect any inflammatory response in the RHOE (Fig. 4D).

**Figure 5:**
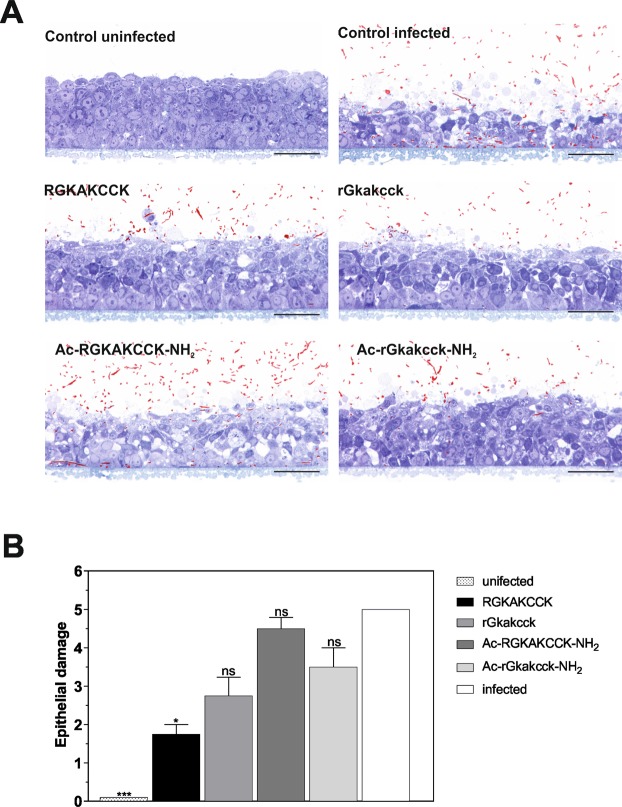
Octapeptides are potential candidates for antimicrobial drug development. (**A**) Reconstituted human oral epithelia were pre-incubated with PBS (control uninfected) or 50 µg/ml of octapeptides as indicated. Subsequently cells were infected with *C*. *albicans* SC5314 (highlighted in red). Representative images are shown (magnification 400x, bar = 100 µm). (**B**) Epithelial damage was evaluated by four independent experts and the combined evaluation (mean +/− SEM, criteria described in methods) is shown. The statistical significance was evaluated by using Kruskal-Wallis test with ns = not significant and *p < 0.05.

Suitability of the octapeptides as novel candidates for antimicrobial drug development was further substantiated in a model or oral candidiasis^26^. In this model a multilayer of RHOE was preincubated with 50 µg/ml (Fig. 5A) or 100 µg/ml octapeptides (Supplementrary Fig. 5) for 1 h before infecting the cells with *C*. *albicans* for 24 h (Fig. 5A). Epithelial damage of RHOEs was quantified by independent experts in a blinded manner on a scale between 0 and 5 (Fig. 5B). Untreated cells infected with *C*. *albicans* displayed evident epithelial damage and cell lysis and detectable fungi (coloured in red) in all layers of the epithelium. However, pre-treatment with RGKAKCCK convincingly reduced the fungal load in the epithelium and ameliorated epithelial damage. Additionally, a histological analysis confirmed the protective effect of RGKAKCCK (Fig. 5B) whereas a pre-treatment with 50 µg/ml rGkakcck moderately improved epithelial damage. In contrast, epithelial damage and a high amount of detectable fungal cells in lower epithelium were observed with a pre-treatment with the modified octapeptides Ac-RGKAKCCK-NH_2_ and Ac-rGkakcck-NH_2_: While *C*. *albicans* cells invaded the whole epithelium with a pre-treatment with Ac-RGKAKCCK-NH_2_, *C*. *albicans* invaded and damaged only the upper epithelium with a pre-treatment with Ac-rGkakcck-NH_2._ Consequently, our eight amino-acid peptide RGKAKCCK of the carboxy-terminus of hBD1 has the best potential to be optimized for topical application against infectious microbes on epithelial surfaces.

## Discussion

So far, no multicellular organism has been identified which does not produce antimicrobial peptides. Even over a long time of evolution those host defense molecules have retained their antimicrobial capacity with only minor resistance mechanisms at the microbial target^15,27^. Thus, an improved knowledge of these antibacterial molecules may help to identify novel targets for antimicrobial therapy^28^. In fact, in the recent years short antimicrobial peptides have gained increased interest as new opportunities for therapeutics^29^. In here, we characterized a carboxy-terminal octapeptide of hBD-1 that was generated after proteolytic digestion by gastro-intestinal proteases and might thus occur *in vivo* in the human gastrointestinal tract.

While reduction of disulphide bridges increases activity of several antimicrobial peptides^11,30,31^, it also increases their susceptibility towards proteolytic digestion. This is especially relevant in the intestinal tract, where duodenal secretions contain high amounts of proteases to facilitate digestion of dietary proteins. However, our observation that a degradation product of hBD-1 retains antibiotic activity indicates effective utilization of a scare resource: we speculate that once the reduced peptides diffuse from the intestinal mucus layer towards the lumen, they can be degraded as soon as they come into contact with intestinal proteases. But instead of being inactivated, degradation products can retain their antimicrobial activity, thereby making the most effective use of these antimicrobial host defense molecules.

While a major shift in antimicrobial activity and activity spectrum can be observed after reduction of the oxidized hBD-1 (Fig. 1D, consistent with^11,12,32^, only minor alterations can be observed after degradation of the reduced peptide. However, on a molar basis the octapeptide does not exhibit the full potency of the full-length peptide, suggesting that the remaining 28 amino acids of hBD-1 hold additional features that enhance and modulate antibiotic activity as, for instance, observed for *Bifidobacterium breve* (Fig. 1D). Remarkably, and in contrast to hBD-1, a reducing environment diminished antimicrobial activity of the tested octapeptides against *E*. *coli* and *C*. *albicans* (Supplementrary Fig. 2). It is possible that due to their small size the peptides need to form dimers or higher-order oligomers to effectively penetrate the microbial cell wall. It is likely that such oligomerization occurs by forming intermolecular disulphide-bridges, thus explaining the strong dependence on cysteine residues for the activity of RGKAKCCK against *E*. *coli* and *C*. *albicans* (Fig. 1E). We could observe that human blood serum can influence bacterial growth and the antimicrobial activity (Supplementrary Fig. 3). Similarly, acidic pH led to lower antimicrobial activity as compared to pH 7.4 (Supplementrary Fig. 2). This is in accordance with previous studies, which could show that antimicrobial peptides can bind to human plasma proteins^33,34^. Thus, further optimization of a potential peptide formulation would be required to employ the octapeptide as a topical skin therapeutic, as human skin has a pH of about 5.5.

The cell envelope is a commonly discussed target for antimicrobial peptides^35^. While the full length hBD-1 targets the bacterial cell wall and entrapped bacteria in net-like structures, the octapeptides seem to have distinct antimicrobial mechanisms (Fig. 2D). Our methods revealed a breakdown of membrane potential and loss of membrane integrity in bacteria. In contrast, treated fungal cells displayed a functional membrane but cytosolic defects (Fig. 3). These data highlight that our octapeptides have diverse antibiotic strategies for different microorganisms. Remarkably, even for the same microbial species, we observed strain specific differences in susceptibility. While the *P*. *aeruginosa* ATCC type strain was not susceptible towards our octapeptides (Figs 1D and 2A), the multi-resistant *P*. *aeruginosa* 4-MRGN strain was susceptible (Fig. 2B). This is in accordance with previous studies, which could show that antibiotic-resistant bacteria show an increased sensitivity against antimicrobial peptides^36^.

Different peptides derived from β-defensins have already been investigated on their antimicrobial activity against bacteria and fungi^37,38^. For instance, 19-mer peptides derived from the carboxy-terminus of hBD-1 or −2 and a 22-mer derived from the terminus of hBD-3 were analyzed on their antibiotic activity. These peptides retained one disulphide bridge and had lethal concentrations in the low micromolar range. With our carboxy-terminal octapeptide we can facilitate synthesis by significantly shortening the amino acid sequence and by omitting the disulphide bridge. Most studies investigating defensin-based peptides have been focused on hBD-3, which is one of the most potent AMPs. By generating different amino-carboxy-terminal peptides, Hoover *et al*. identified several carboxy-terminal peptides with 9 to 14 amino acids having activity against *E*. *coli* or *P*. *aeruginosa*, but not against *S*. *aureus*^39^. In these peptides, cysteine residues were replaced by serine residues, which we found to be also crucial for activity in our peptides. Also, Reynold *et al*. described that antimicrobial activity of hBD-3 was mainly localized in the amino-terminal half^40^. Similar to our results, they reported that distinct amino acids are important for activity against different strains, suggesting that the strain-selectivity of such peptides can be modulated by varying the sequence.

To be utilized as potential antimicrobial molecules, it has to be excluded that the generated peptides provoke resistance of the treated microorganism. By using a host-derived antimicrobial peptide as therapeutic, this is of major importance, as resistance or cross-resistance towards other AMPs might be fatal for the host. While further testings are required to determine whether our identified peptides provoke such resistance, we believe that the octapeptide can serve as a backbone structure that could be optimized to enhance and/or specialize its activity and to diverge from its natural structure, which would decrease the risk of resistance induction. For example, due to their difference in activity against *E*. *coli* and *C*. *albicans*, optimization of the peptides RGKAKACK and RGKAKCAK (Fig. 1E) could generate a peptide that is effective against *E*. *coli*, but not against *C*. *albicans*.

In conclusion, we identified that the host can broaden its antimicrobial arsenal by generating several antibiotic molecules from the AMP hBD-1, depending on its redox state and proteolytic degradation. We believe that this strategy can be therapeutically exploited and that our identified hBD-1 derived carboxy-terminal peptides can be optimized for topical application against bacterial or fungal infections.

## Materials and Methods

### Bacterial and fungal strains

Bacterial strains (*Bifidobacterium adolescentis* Ni,29c (clinical isolate), *Bifidobacterium breve* (from probiotic VSL#3) and *Streptococcous salivarius ssp*. *thermophiles* DSM 20617 were obtained from Ardeypharm (Germany). *Escherichia coli* ATCC 25922, *Escherichia coli* K12, *Pseudomonas aeruginosa* ATCC 27853, *Candida albicans* ATCC 10231, *Enterococcus faecalis* ATCC 29212 as well as antibiotic-resistant clinical isolates of *Acinetobacter baumannii*, *Enterococcus faecalis*, *Enterococcus faecium*, *Klebsiella pneumoniae*, *Escherichia coli*, *Staphylococcus aureus* and *Pseudomonas aeruginosa* were provided by the Department for Laboratory Medicine at Robert-Bosch-Hospital Stuttgart, Germany. *Candida albicans* SC5314 was obtained from Salomé LeibundGut-Landmann (Institute of Immunology, Vetsuisse Faculty, University of Zürich, Switzerland).

### Peptides

Carboxy-terminal octapeptides were chemically synthesized by EMC Microcollections (Tuebingen, Germany) and purified by precipitation. The oxidized peptides were obtained from Peptide Institute (Japan). All peptides were dissolved in 0.01% acetic acid.

### Protease digestion and Matrix-assisted laser desorption/ionization mass spectrometry (MALDI-MS)

2 µg of oxidized or reduced hBD-1 were digested with pepsin or chymotrypsin at a protease: peptide ratio of 1:20 in HCl-acidified water, pH 3 (pepsin) or 10 mM sodium phosphate, pH 7.4 (chymotrypsin) for 90 min. Human duodenal secretion (pH 6.5–7) was taken during a routine gastroscopy by rinsing the duodenum with saline. Oxidized and reduced hBD-1 were incubated with human duodenal secretion for 30 min at 37 °C. As a control both peptides were incubated with 0.9% NaCl. Peptides were enriched with ZipTip (Millipore), co-crystallized with a-cyano-4-hydroxy cinnamic acid and analyzed with an ultraflex TOF/TOF machine (Bruker, Germany).

### Radial diffusion assay

Antimicrobial radial diffusion assay was modified from reference^17^ and performed as described earlier^11^. Briefly, microorganisms were cultivated (anaerobic bacteria with AnaeroGen, Oxoid, UK) for up to 18 hours in liquid TSB medium. Log-phase cultures were washed and diluted to 4 × 10^6^ colony forming units in 10 ml agar. Incubation was carried out in 10 ml of 10 mM sodium phosphate, either pH 7.4 or 5.7, containing 0.3 mg/ml of TSB powder and 1% (w/v) low EEO-agarose (AppliChem) with 0 or 1 mM dithiothreitol (DTT, Sigma-Aldrich) under anaerobic or aerobic conditions for three hours. 1 or 4 µg of synthetic, oxidized hBD-1 (Peptide Institute, Japan) and 1 or 4 µg of synthetic peptides (EMC Microcollections, Tuebingen) were filled into small punched wells in a final volume of 4 µl. This concentrated peptide solution dilutes while diffusing into the gel, thereby generating concentration-dependent, round-shaped inhibition zones when killing immobilized microorganisms.

An overlay-gel containing 6% (w/v) TSB powder, 1% agarose and 10 mM sodium phosphate buffer without DTT was poured onto the plates and after incubation for up to 48 h at 37 °C the diameter of inhibition zones was measured. Experiments were repeated at least three times; mean + SEM is shown.

### Microdilution broth assay

To differentiate between microbistatic and microbicidal activity we performed a broth microdilution assay. For that, *E*. *coli* ATCC25922 bacteria were incubated overnight at 37 °C, 150 rpm. *C*. *albicans* ATCC 10231 was grown at 30 °C overnight, 150 rpm in liquid TSB. Cells were collected by centrifugation (2500 rpm, 10 min, 4 °C), washed twice and resuspended in 10 mM sodium phosphate buffer containing 1% (w/v) TSB broth. Required *C*. *albicans* cell density was adjusted using a hemocytometer. For bacteria the optical density of OD_600nm_ = 0.1 was determined. Approximately 5 × 10^5^ CFU/ml bacteria or fungi were mixed with indicated peptide concentrations (1.25–200 µg/ml) in a final volume of 100 µl in 10 mM sodium phosphate buffer containing 1% (w/v) TSB broth and incubated for 2 hours at 37 °C. After incubation 10 µl per well were plated on LB-/ or YPD-agar plates to determine the CFU/ml. After that 100 µl of 6% TSB (w/v) were added and absorbance was measured at 600 nm (Tecan, Switzerland) and monitored for 18 hours. Growth relative to the positive control in % was plotted against peptide concentration. Experiments were carried out at least three times; mean ± SEM is shown.

### Flow cytometry assay

Approximately 1.5 × 10^6^ CFU log-phase bacteria or overnight cultured *C*. *albicans* ATCC 10231 were used in a final volume of 100 µl TSB (1:6 diluted H2O). We added peptides in concentrations 50 and 100 µg/ml in a final volume of 10 µl and incubated theses suspensions for 1 h at 37 °C. Subsequently 2 µl of membrane potential sensitive dye DiBAC_4_(3) [bis-[1,3-dibutylbarituric acid)trimethine oxonol] (Thermo Scientific, USA) (50 µg/ml) or Propidium Iodide (Thermo Scientific, USA) (50 µg/ml) were added and incubated for 10 min at room temperature. Then samples were centrifuged (5 min, 7000 rpm, RT) and re-suspended in 300 µl PBS. The percentage of fluorescent positive cells was determined using Canto II flow cytometer (BD Bioscience) with DIVA software (BD Bioscience) as described earlier^41^. Experiments were repeated at least three times and mean +/− SEM is shown.

### Transmission electron microscopy

Approximately 1 × 10^8^ CFU of *E*. *coli* ATCC 25922 and *C*. *albicans* ATCC 10231 were incubated with 400 µg/ml peptides or control solution for 2 hours at 37 °C. Treated microorganism were fixed with Karnovsky’s fixative (3% Paraformaldehyd, 3.6% Glutaraldehyd, pH 7,2) and embedded in 3.5% agarose at 37 °C, coagulated at room temperature, and fixed again in Karnovsky’s fixative. Post-fixed samples (1% OsO_4_, 1 h) were rinsed with distilled water, block-stained with uranyl acetate (2% in distilled water), dehydrated in alcohol (stepwise 30–96%), immersed in propylene oxide and embedded in glycine ether (polymerized 48 h at 60 C, Serva, Heidelberg). Ultra-thin sections were examined with a LIBRA 120 (Carl Zeiss AG, Oberkochen) at 120 kV.

### Metabolic activity Assay

To assess the metobalic activity of Caco-2 cells we used the WST-1 Cell Proliferation Reagent (Roche, Germany). Briefly, 1 × 10^5^ Cells /ml were seeded and incubated with 100 µg/ml or 200 µg/ml octapeptides for 24 h at 37 °C, 5% CO_2_. After incubation the supernatant was removed and cells were washed with PBS and incubated with 20 µl Cell Proliferation Reagent WST-1 for 1 h 37 °C, 5% CO_2_. Finally the absorbance was measured at 450 nm and 620 nm. Experiments were repeated three times, mean+/− SEM.

### Hemolytic Activity of antimicrobial peptides

An hemolytic activity assay for testing antimicrobial peptides was performed as described earlier^42^. Briefly, 150 µl of melittin (5 µM) was added to the positive control wells and incubated overnight. On the next day 1 ml blood was added to 3 ml PBS, mixed gently and centrifuged for 8 min, 700 × g. The supernatant was discarded and cells were re-suspended in 4 ml PBS and centrifuged again. After removing the supernatant, cells were centrifuged for 8 min at 1000 × g. Supernatant was discarded. For each well we used 75 µl of 1% Red blood cell (RBC) suspension in PBS. RBC suspension was mixed with indicated peptide concentration (2.5–200 µg/ml) in a final volume of 150 µl and incubated for 1 h at 37 °C. Finally the plate was centrifuged at 1000 × g for 10 min and 60 µl of supernatant was quickly transferred into a new plate. The absorbance was measured by 405 nm and 540 nm. Hemolytic activity was plotted relative to the 0.2% Triton X-100. Experiments were repeated three times; mean +/− SEM is shown.

### Culture of a model human oral epithelium

1 × 10^6^ TR146 cells (derived from a squamous cell carcinoma of the buccal mucosa; SkinEthic, France) were seeded into polycarbonate plastic inserts (Millipore) in DMEM (Lonza) and cultured for 8 days to form a multilayered epithelium. Medium in the wells (basal) and in the inserts (apical) was changed daily. On day 5 (airlift) medium was aspired from the apical side and cells were fed from the basal side for the rest of culture time in 6-well-plates. No antibiotics were used for the entire time of culture and the experiments^43^. The octapeptides were diluted to 100 µg/ml in 0.01% acetic acid and 50 µl of these dilutions were applied to the apical side of the model epithelia and incubated for 24 h. The supernatant was used for cytotoxicity assays and Enzyme-linked immunosorbent assay.

### LDH-Cytotoxicity Assay

To analyze the damage of the epithelial cells caused by octapeptides, release of lactate dehydrogenase (LDH) into the supernatant of RHOEs was quantified, using the cytotoxicity detection kit with L-LDH solution as standard, according to the manufacturer’s instructions (Roche, Germany). Supernatants of RHOEs were analyzed 24 h post infection.

### Enzyme-linked immunosorbent assay

Interleukin 8 (IL8) and IL1α were quantified in the supernatants of RHOEs using DuoSet ELISA-Kits (RnD Systems, US) according to the manufacturer’s instructions. Further details are explained above (Culture of a model human oral epithelium).

### Infection of model human oral epithelium with *C*. *albicans*

Octapeptides were diluted to 50 µg/ml in PBS, or an equivalent volume of 0.01% acetic acid were applied to the apical side of the RHOEs and incubated for 1 h. *C*. *albicans* SC5314 was synchronized as described previously^26^. Yeast cells were washed three times in PBS and 1 × 10^5^ CFU were used for infection of pre-treated RHEs. After 24 h RHEs were fixed with Karnovsky’s fixative. Post-fixed samples (1% OsO_4_, 1 h) were rinsed with distilled water, block-stained with uranyl acetate (2% in distilled water), dehydrated in alcohol (stepwise 30–96%), immersed in propylene oxide and embedded in glycide ether (polymerized 48 h at 60 °C, Serva, Heidelberg). Semi-thin sections were stained with toluidine blue and examined with a Nikon Eclipse 80i light microscope (magnification 1:400). Afterwards, fungal cells were simply coloured in red. Epithelial damage was evaluated by two independent experts in a blinded manner on a scale between 0 and 5 with 0 = intact epithelia, 1 = damage only in topmost cell layer; 2 = damage in top third of epithelium; 3 = damage in top half of epithelium; 4 = damage in all cell layers except lowermost; 5 = damage in all epithelial layers.

### Ethics statement

The study protocol was previously approved by the Ethical Committee of the University Hospital, Tuebingen, Germany. Patients and controls who were included in this study all gave their written and informed consent after the study purpose, samples procedure, and potential adjunctive risks were explained. All experiments were conducted in accordance with the relevant guidelines and regulations.

### Statistical analysis

Results are presented as mean +/− SEM from at least three independent experiments. Statistical analysis was performed using GraphPad Prism 7.03. The Data were not normally distributed and a Kruskal-Wallis test was performed. *Indicate statistically signifant differences compared to infected control. P-values, showing the statistical significance, were displayed by asterisks: p > 0.05 = ns; *p ≤ 0.05; **p ≤ 0.01; ***p ≤ 0.001; ****p ≤ 0.0001.

## Supplementary information

Supplementary Material
