# Supplementary material for: Proteolytic Degradation of reduced Human Beta Defensin 1 generates a Novel Antibiotic Octapeptide
Source: Sci Rep. 2019 Mar 6;9:3640. doi: 10.1038/s41598-019-40216-2 (PMC6403363; doi:10.1038/s41598-019-40216-2)
Supplement: Supplementary file 1 — Supplementary Material [file 41598_2019_40216_MOESM1_ESM.docx]

**Proteolytic Degradation of reduced Human Beta Defensin 1 generates a Novel Antibiotic Octapeptide**

**Authors:** Judith Wendler ^1,#,^, Bjoern O. Schroeder ^2,6,#^, Dirk Ehmann^1^, Louis Köninger^1^, Daniela Mailänder-Sánchez^1^, Christina Lemberg^4,7^, Stephanie Wanner^4,5^, Martin Schaller^4^, Eduard F. Stange^2^, Nisar P. Malek^1^ , Christopher Weidenmaier^5^, Salomé LeibundGut-Landmann^3^, Jan Wehkamp^1^

^#^these authors have contributed equally

**Affiliations:**

^1^ Department of Internal Medicine 1, University Hospital Tuebingen, Germany

^2^ Dr. Margarete Fischer-Bosch-Institute of Clinical Pharmacology, Stuttgart and University of Tuebingen, Germany

^3^ Institute of Immunology, Vetsuisse Faculty, University of Zürich, Switzerland

^4^ Institute of Dermatology, University Hospital Tuebingen, Germany

^5^ Institute of Medical Microbiology and Hygiene, University Hospital Tuebingen, Germany

^6^ Present address: Wallenberg Laboratory, University of Gothenburg, Sweden

^7^Present address: Institute of Immunology, Vetsuisse Faculty, University of Zürich, Zürich, Switzerland

*to whom correspondence should be addressed: [jan.wehkamp@med.uni-tuebingen.de](mailto:jan.wehkamp@med.uni-tuebingen.de)

**Supplementary Material**

**
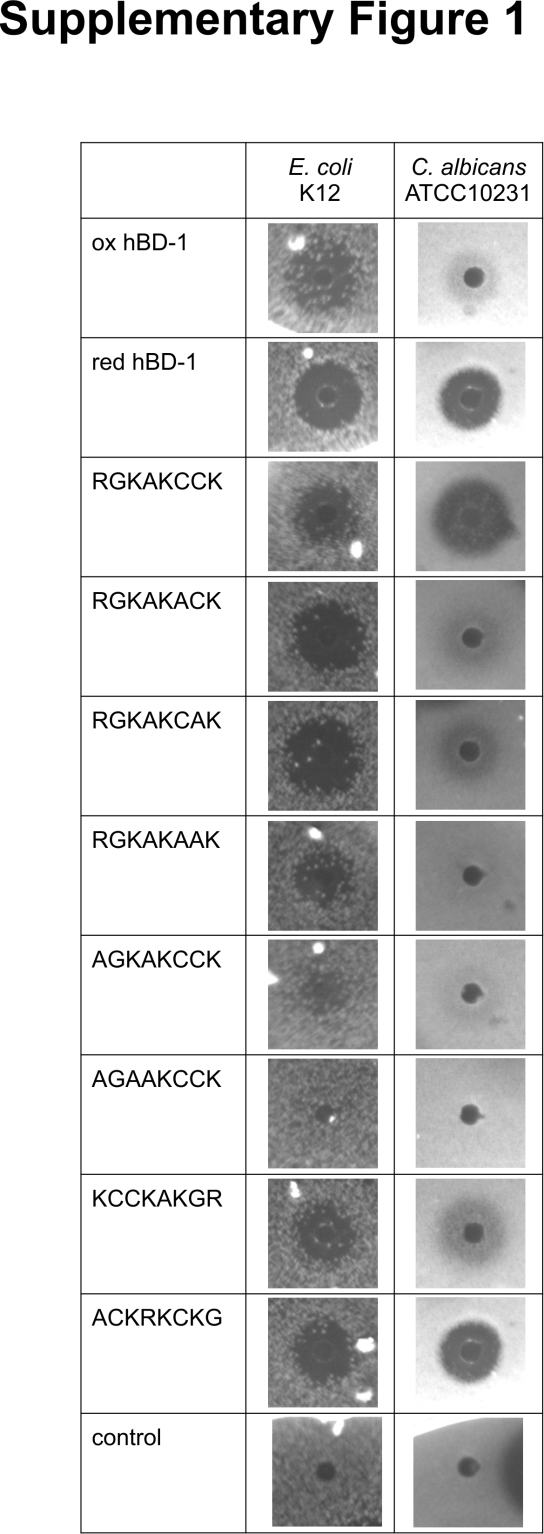
**

**Supp. Fig. 1: Representative Images of radial diffusion assays.**

Different peptides (4 µg) were tested in an antimicrobial diffusion assay against several microbial strains. Diameter of inhibition zones indicates antimicrobial activity; a diameter of 2.5 mm is the diameter of an empty well. Representative images are shown.

**
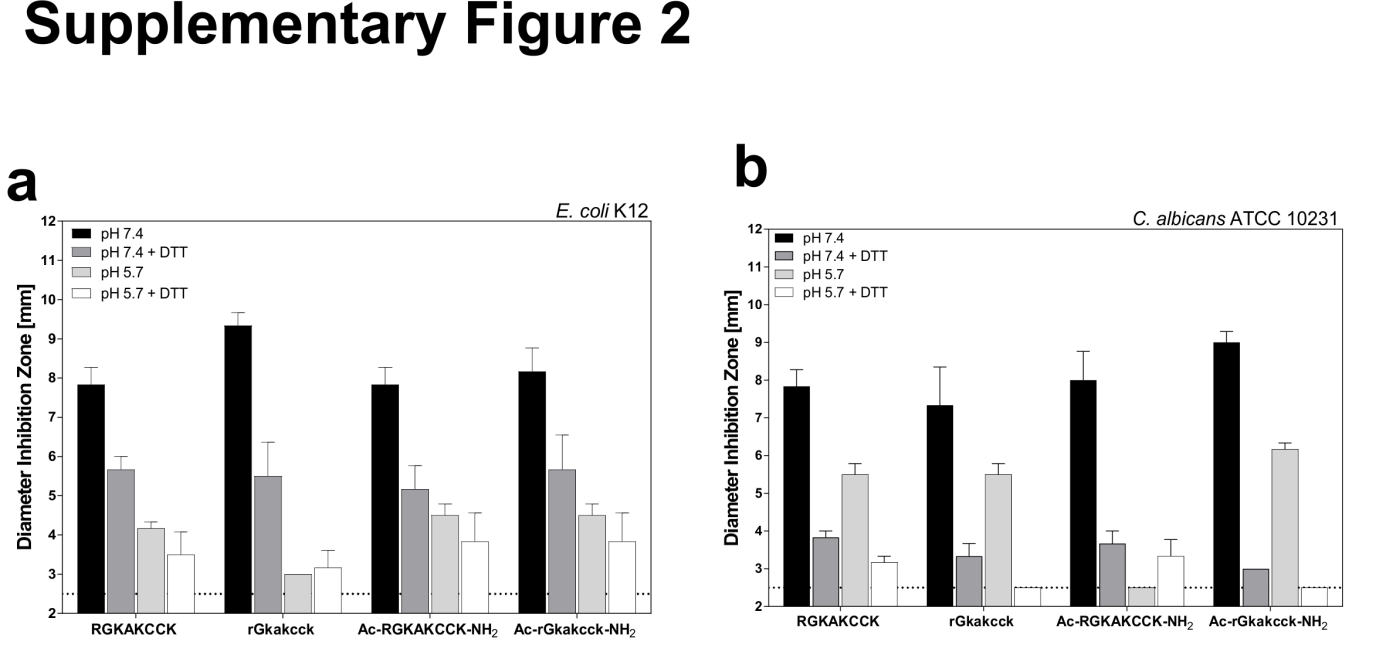
**

**Supp. Fig. 2: Chemical environment influences antimicrobial activity of RGKAKCCK and its modified variants.** The carboxy-terminal octapeptide RGKAKCCK was stabilized at its termini by acetylation of the amino-terminus and amidation of the carboxy-terminus (Ac-RGKAKCCK-NH_2_). Peptides (4 µg) were investigated on their antimicrobial activity against **(a)** *E.coli* K12 *and* **(b)** *C. albicans* ATCC 10231*.* Radial diffusion assay was carried out under standard conditions (black), reducing conditions (dark grey) or by using an acidic pH without DTT (light grey) and with DTT (transparent) . Letters indicate amino acid one-letter code. Diameter of inhibition zones indicates antimicrobial activity; a diameter of 2.5 mm (dotted line) is the diameter of an empty well. Experiments were carried out at least three times, mean + SEM is shown.


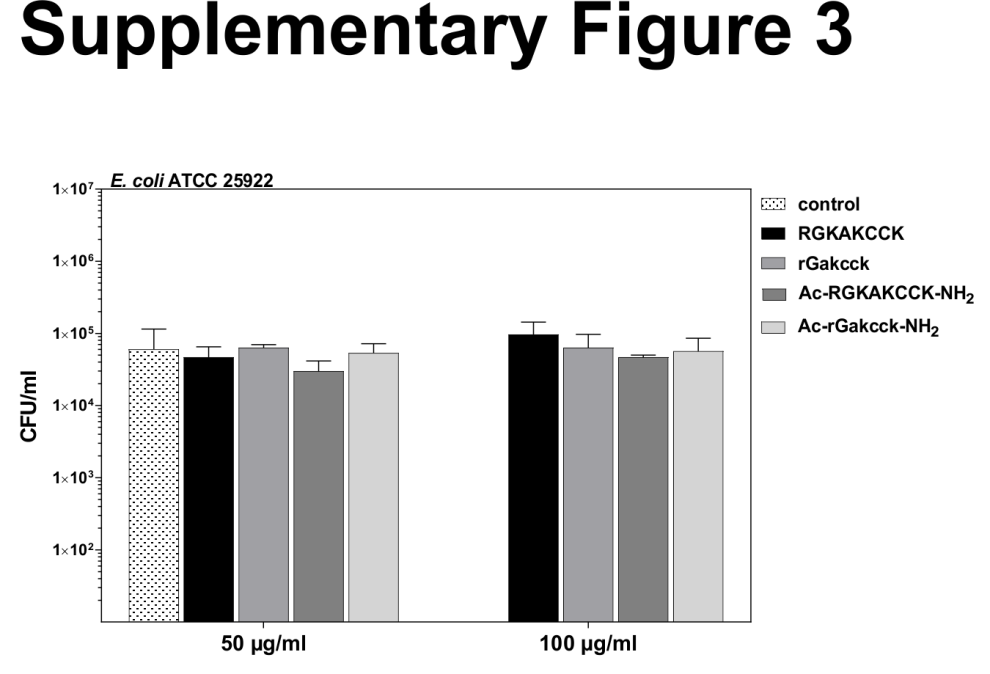


**Supp. Fig. 3: Presence of human blood serum influences antimicrobial activity of octapeptide variants.**

Two different concentrations of octapeptides were tested in a turbidity liquid assay against *E. coli* ATCC 25922. Peptides were incubated in human blood serum with tested microorganism. After 2h aliquots were plated on agar plates and colony forming units (CFUs) were calculated the next day. Data are presented as mean +/- SEM of at least three independent experiments.

.


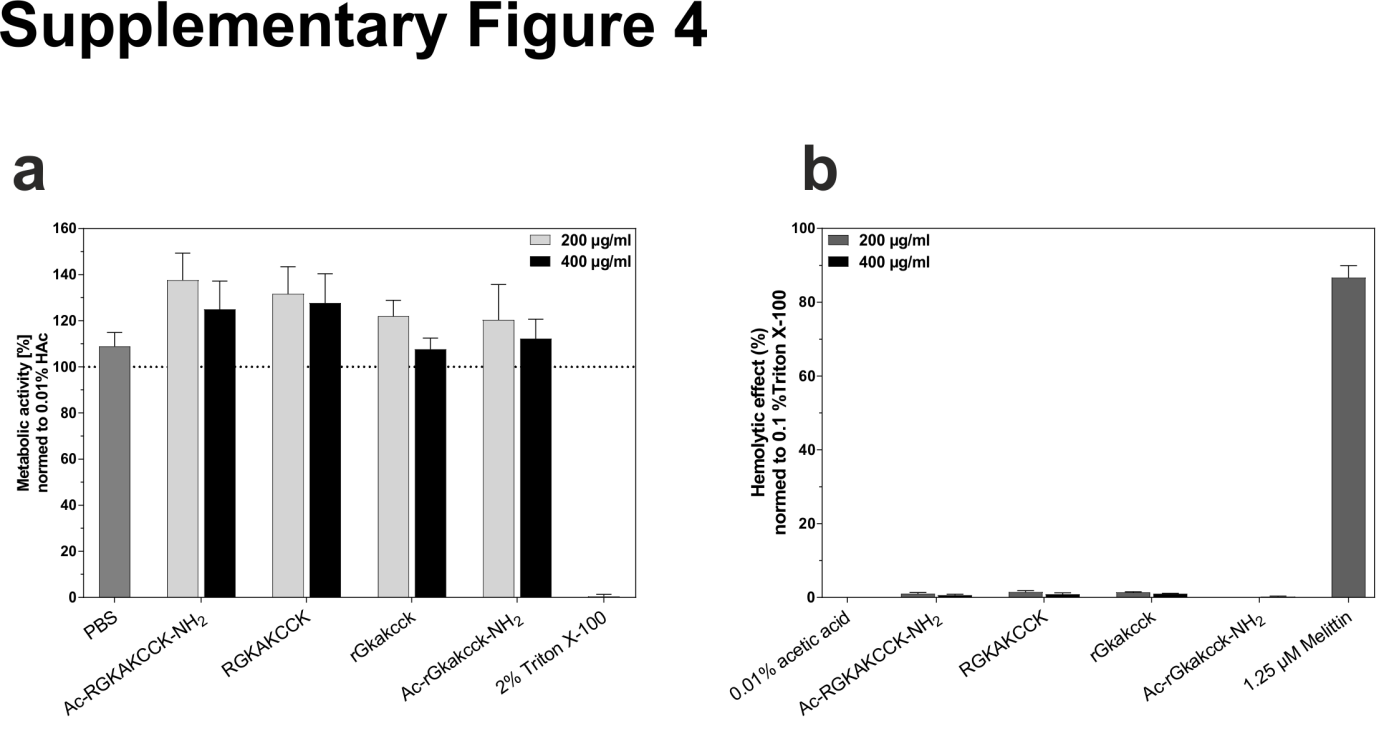


**Supp. Fig. 4: Modified octapeptides are not cytotoxic in higher concentrations.**

200 µg/ml and 400 µg/ml of different octapeptides were tested in two different cytotoxicity assays. (a) A WST-1 based test with human intestinal epithelial cell lines CaCo-2 and (b) Hemolytic Activity assay ^37^ with human red blood cells were used. We used 2% Triton-X-100 and 1.25 µM Melittin as positive controls. Data are presented as mean /- SEM of at least three independent experiments.


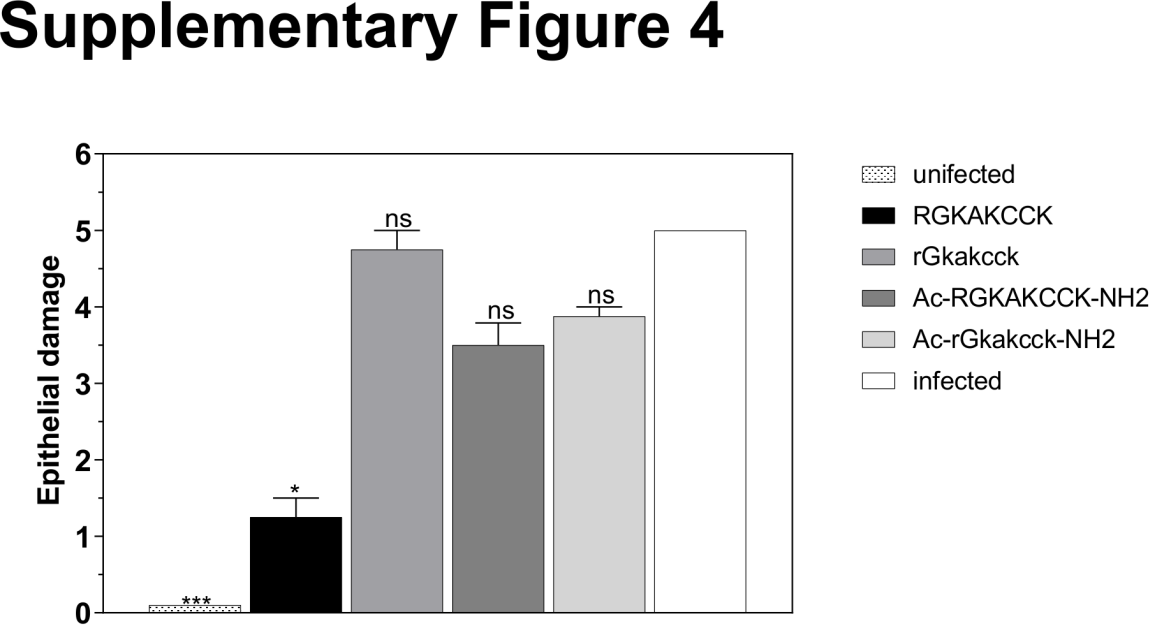


**Supp. Fig. 4: Efficacy of octapeptides in an oral infection epithelia model.**

Reconstituted human oral epithelia were pre-incubated with PBS (control uninfected) or 100 µg/ml of octapeptides as indicated. Subsequently cells were infected with *C. albicans* SC5314. Epithelial damage was evaluated by four independent experts and the combined evaluation (mean +/- SEM, criteria described in methods) is shown. Data are presented as mean /- SEM of at least three independent experiments. The statistic was evaluated by using Kruskal-Wallis test with ns = not significant and *= p <0.05.
